# Molecular Flexibility-Controlled Ion Solvation and Electrode Reaction Kinetics in Sulfite-Based Lithium-Ion Battery Electrolytes

**DOI:** 10.1021/acs.jpcb.5c08092

**Published:** 2026-02-19

**Authors:** Misa Yamashita, Saki Sawayama, Kenta Fujii

**Affiliations:** Graduate School of Sciences and Technology for Innovation, 13150 Yamaguchi University, 2-16-1 Tokiwadai, Ube, Yamaguchi 755-8611, Japan

## Abstract

The effects of solvent molecular flexibility on Li^+^ solvation and graphite electrode kinetics were investigated in lithium-ion battery (LIB) electrolytes using dimethyl sulfite (DMS), a linear sulfite solvent with high molecular flexibility. Raman spectroscopy revealed that, in the dilute solutions, Li^+^ ions are solvated by three DMS molecules to form Li­(DMS)_3_^+^ complexes, whereas the corresponding cyclic sulfite solvent, ethylene sulfite (ES), forms conventional four-coordinate Li­(ES)_4_^+^ complexes. Density functional theory (DFT) calculations revealed that the Li­(DMS)_3_^+^ complex consists of two monodentate DMS molecules and one bidentate DMS molecule, the latter adopting the trans–trans (TT) conformer, which is thermodynamically unfavorable in the bulk phase, but becomes stabilized within the Li^+^ solvation shell due to the strong electrostatic field of the Li^+^ ion. As a result, the Li­(DMS)_3_^+^ complex is energetically less stable than the conventional four-coordinate Li­(ES)_4_^+^ complex. In the highly concentrated region, Li^+^ ions formed similar ionically ordered structures interconnected through bis­(fluorosulfonyl)­amide (FSA) anions in both DMS and ES electrolytes. In the electrode reaction, the dilute DMS electrolyte exhibited an exceptionally low activation energy for Li^+^ insertion at the graphite electrode, attributed to the easier desolvation of weakly coordinated Li^+^ species. However, its reductive instability led to DMS-derived SEI films with poor stability and rapid capacity degradation. In contrast, the highly concentrated DMS electrolyte produced stable FSA-derived solid–electrolyte interphase (SEI) films and improved cycling stability, albeit with higher activation energy due to dominant Li^+^–anion interactions. These results provide molecular-level insights into how solvent flexibility governs Li^+^ coordination structures and electrode reaction kinetics in LIB electrolytes.

## Graphical abstract

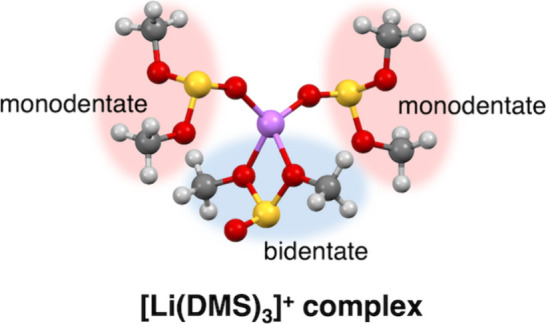

## Introduction

Electrolyte solvents play a central role in the operation of lithium-ion batteries (LIBs), functioning not only as media for ion transport but also as chemical environments that directly govern the solvation state of Li^+^ ions and thereby control the electrode reactions. In particular, for graphite, the most widely used anode material for LIBs, it has been established that the mechanism of Li^+^ insertion and deinsertion strongly correlates with the desolvation process of Li^+^ at the electrode–electrolyte interface.^1,2^ In conventional carbonate-based electrolytes, consisting of mixtures of cyclic and linear carbonates such as ethylene carbonate (EC) and dimethyl carbonate (DMC), the activation energy for Li^+^ insertion is approximately 53–59 kJ mol^–1^, a value that is essentially independent of the electrode material and is well established to reflect the Li^+^ desolvation energy.^2–5^ These observations indicate that the key factor controlling interfacial kinetics is not the behavior of Li^+^ within the electrode but rather the nature of Li^+^–solvent interactions in the electrolyte solution.

In conventional carbonate-based electrolytes, cyclic carbonates such as ethylene carbonate (EC) and vinylene carbonate (VC) play indispensable roles by serving not only as high-permittivity solvent components but also as key additives that form stable and passivating solid–electrolyte interphase (SEI) films on graphite anodes.^6–9^ However, their high melting points or high viscosities at ambient temperature limit their use as sole solvent components. In contrast, ethylene sulfite (ES), a cyclic sulfite solvent with a chemical structure analogous to EC, is a low-viscosity liquid at room temperature and is capable of forming highly stable SEI films on graphite electrodes.^10,11^ We have recently reported that ES-based electrolytes containing lithium bis­(fluorosulfonyl)­amide (LiFSA) enable reversible and stable Li^+^ insertion and deinsertion across a wide concentration range, from dilute to highly concentrated conditions.^12^ Importantly, the Li^+^ solvation structure changes continuously with increasing salt concentration: (1) in the dilute region (Li salt concentration, *c*_Li_ < 1 M), Li^+^ forms conventional four-coordinate solvation complexes, Li­(ES)_4_^+^; (2) at intermediate concentrations (∼2.5 M), contact ion pairs emerge; and (3) in the highly concentrated region (>3 M), ionically ordered structures (or ionic aggregates) appear, in which Li^+^ ions are interconnected via FSA anions. Although these structural changes in Li^+^ solvation are expected to strongly influence electrode reaction mechanisms, particularly the Li^+^ desolvation process, the molecular-level relationship between solvation and electrode reaction properties has remained largely unexplored.

In understanding and controlling metal–ion solvation in solution, traditional solvent parameters such as electron-donating ability, represented by the Gutmann donor number, and steric factors associated with solvent size are widely used.^13–16^ However, in addition to these parameters, the molecular flexibility of solvent molecules or counteranions whose ability to undergo internal conformational changes, has increasingly been recognized as a factor that can significantly modulate ion solvation structures.^17–20^ Such molecular flexibility can alter the coordination environment around Li^+^ ions and directly influence ion-transport properties as well as electrode reaction characteristics. We recently reported a unique Li^+^ solvation motif arising from the conformational flexibility of the FSA^–^ anion in ionic liquid (IL) electrolytes.^20^ In bulk ILs without Li salt, the FSA^–^ anion exists in equilibrium between cis- and trans-FSA conformers, in which the two F atoms are positioned at cis and trans orientations relative to the S–N–S plane; the activation barrier for interconversion between these conformers is sufficiently low that FSA^–^ exhibits high molecular flexibility.^21,22^ This intramolecular flexibility persists even within the Li^+^ solvation shell: a bidentate-coordinated FSA^–^, a coordination mode commonly observed in imide-type anions such as bis­(trifluoromethanesulfonyl)­amide (TFSA^–^),^23–25^ can change its conformation to yield a monodentate coordination under the strong electrostatic field of Li^+^.^20,26^ The monodentate FSA^–^ interacts with Li^+^ through only a single O atom, resulting in substantially weaker Li^+^–FSA^–^ interaction compared to chelating bidentate FSA^–^, and this weakened interaction directly facilitates easier Li^+^ desolvation, yielding a lower activation energy and kinetically favorable Li^+^ insertion processes.^20^

In this study, we focused on the molecular flexibility of solvent molecules and selected dimethyl sulfite (DMS, (CH_3_O)_2_SO), a linear sulfite solvent with the same chemical composition as cyclic ES, as a model system to elucidate how conformational freedom is associated with Li^+^ solvation structures and electrochemical properties. In the context of this study, the term “molecular flexibility” is used in a descriptive sense to denote the ability of solvent molecules to adopt multiple intramolecular conformations, which allows for structural diversity in local coordination environments upon Li^+^ solvation. DMS is known to possess multiple conformers, in which the –OCH_3_ groups adopt gauche or trans orientations relative to the O–S–O plane, resulting in higher intramolecular conformational diversity than rigid cyclic ES.^27^ By systematically varying the Li salt concentration in LiFSA/DMS electrolytes from dilute to highly concentrated conditions, we clarified Li^+^ solvation structures and their correlation with electrode reaction behavior. Our findings reveal that, in the dilute region, solvent molecular flexibility is closely associated with the Li^+^ coordination environment and the resulting desolvation behavior and electrode reaction kinetics, whereas in the highly concentrated region, similar ionically ordered structures are formed in both DMS- and ES-based electrolytes, leading to similar electrochemical characteristics.

## Experimental Section

### Materials

LiFSA salt (Kanto Chemical, Battery grade) was vacuum-dried at 373 K for 24 h before use. DMS (Tokyo Chemical Industry Co.) and ES (Sigma-Aldrich) were dehydrated over molecular sieves (3 Å) for several days and vacuum-distilled before use. The sample electrolyte solutions were prepared at given salt concentrations (*c*_Li_/M) in an Ar-filled glovebox. The Li-salt-to-solvent molar ratio and solution density (*d*/g cm^–3^, at 298 K) for the solutions (*c*_Li_ = 0–4.9 M) are listed in Table S1. The water content of the solutions was less than 50 ppm, as determined by Karl Fischer titration.

### Experimental and Computational Methods

Raman spectroscopy was performed using a dispersion-type Raman spectrometer (NRS-3100, JASCO) equipped with a 532 nm laser at 298 K. Each sample solution was sealed in a quartz cell, and spectra were recorded with an optical resolution of 4.0 cm^–1^. The obtained Raman spectra were deconvoluted into individual bands by nonlinear least-squares fitting using a pseudo-Voigt function.^28–30^ Likewise, the integrated intensity of bound DMS in the Li-ion solvation shell is expressed as *I*_b_ = *J*_b_ *c*_b_. From the mass balance relation *c*_f_ = *c*_T_ – *c*_b_ = *c*_T_ – *n*_DMS_ *c*_Li_, where *c*_T_ and *n*_DMS_ denote the total solvent concentration and solvation number, respectively, the following equation is obtained:

$$I_{f}/c_{T}=-n_{\mathrm{DMS}}J_{f}\left(c_{\mathrm{Li}}/c_{T}\right)+J_{f}$$ 1

A plot of *I*_f_/*c*_T_ versus *c*_Li_/*c*_T_ gives a straight line with slope α (= – *n*_DMS_ *J*_f_) and intercept β (= *J*_f_), from which the solvation number is determined by *n*_DMS_ = −α/β. Cyclic voltammetry (CV, HZ-5000; Hokuto Denko) was carried out using a three-electrode cell with a graphite working electrode (1.6 mA h cm^–2^, 0.78 cm^2^; Piotrek) and Li foil as both counter and reference electrodes (3.75 and 0.30 cm^2^, respectively). The scan rate was 0.1 mV s^–1^. Electrochemical AC impedance spectroscopy was performed using a potentiostat/galvanostat (SP-150; BioLogic). For temperature-dependent measurements, impedance spectra were collected over a frequency range from 20 mHz to 1.0 MHz using a three-electrode cell at a fixed electrode potential of 0.1 V (vs Li/Li^+^) under controlled temperatures ranging from 278 to 338 K. In contrast, for potential-dependent measurements, the lower frequency limit was extended to 4 mHz in order to more clearly capture low-frequency impedance responses associated with Li^+^ insertion processes. The impedance spectra obtained for the temperature-dependent measurements were analyzed using an equivalent circuit model commonly applied to graphite electrodes undergoing Li^+^ insertion reactions. The analysis was restricted to the frequency range where stable and reproducible impedance responses were obtained. In this analysis, the resistance component appearing in the low-frequency region was extracted and defined as the low-frequency resistance (*R*_LF_). The extracted *R*_LF_ values were used for determining the apparent activation energy (*E*_a_app_). Ionic conductivity was determined by AC impedance spectroscopy using a potentiostat/galvanostat over the frequency range of 1.0 Hz to 1.0 MHz at 278–338 K. The measurements were carried out using a two-electrode cell composed of stainless-steel electrodes (SUS316, 8.4 mm diameter), with the electrode spacing fixed at 1.4 mm by a Teflon spacer. Charge–discharge cycling tests were performed using CR2032-type coin cells composed of a graphite working electrode (0.78 cm^2^) and a Li-metal counter electrode (1.54 cm^2^). The cells were charged and discharged at 0.1 C, corresponding to a current density of 0.16 mA cm^–2^. Density functional theory (DFT) calculations were performed using Gaussian 09.^31^ The geometries of isolated DMS molecules and Li-ion complexes were fully optimized at the B3LYP/6–311+G** level, followed by normal frequency analysis to ensure that no imaginary frequencies were present. Binding energies (Δ*E*_bind_) for the Li-ion complexes were evaluated as the difference between the electronic energy of the optimized complex and the sum of the energies of the isolated Li^+^ ion and *n* solvent molecules according to Δ*E*_bind_ = *E*_SCF_(complex) – *E*_SCF_(Li^+^) – *nE*_SCF_(solvent), where *n* is the number of coordinated solvent molecules, and corrected for basis-set superposition error using the counterpoise method.^32^

## Results and Discussion

### Li-ion Solvation in DMS-based Electrolytes

Figure 1a shows the Raman spectra of LiFSA/DMS electrolytes as a function of Li salt concentration (*c*_Li_). At *c*_Li_ = 0 M (neat DMS, bold black line in the figure), a prominent peak appears around 580 cm^–1^, which is attributed to the symmetric S–O stretching or out-of-plane S=O bending vibrations (ν­(S–O) and γ­(S=O), respectively).^27^ This spectral feature is closely related to the structural characteristics of the DMS molecule. The DMS molecule is known to exhibit a conformational equilibrium between the gauche–gauche (GG) and gauche–trans (GT) forms in solution due to its high internal flexibility. Indeed, previous DFT calculations have shown that the energy difference between the GG and GT conformers is as small as 0.8 kJ mol^–1^.^27^ Peak deconvolution of the Raman spectrum of neat DMS revealed that it can be reproduced by two components (578 and 580 cm^–1^). These components are consistent with the relative positions of the theoretical Raman bands calculated for the GG and GT conformers and are therefore used as supportive information for the assignment (Figure S1). As the Li salt concentration (*c*_Li_) increased up to 2.0 M, the peak intensity at around 580 cm^–1^, which corresponds to the DMS molecules in the bulk phase (free DMS), gradually decreased. In contrast, a new peak appeared on the higher-frequency side at approximately 590 cm^–1^, and an apparent isosbestic point was observed near 585 cm^–1^ during this spectral variation. This new peak is attributed to DMS molecules coordinated to Li^+^ ions (bound DMS). Consistently, in various organic electrolyte systems, solvent molecules coordinated to Li^+^ are known to exhibit Raman bands shifted toward higher frequencies,^12,26,33–36^ which supports the assignment of this new peak to Li^+^-bound DMS. Upon further increasing *c*_Li_ into the concentrated region (3.0–4.9 M, red lines in Figure 1a), the Raman spectra deviated from the isosbestic point and shifted further toward higher frequency side. Such behavior is commonly observed in highly concentrated electrolytes^12,20,33^ and suggests the formation of ionic aggregates in which Li^+^ ions are coordinated with both solvent molecules and counteranions, leading to ordered Li-ion structures interconnected through anions.

**Figure 1:**
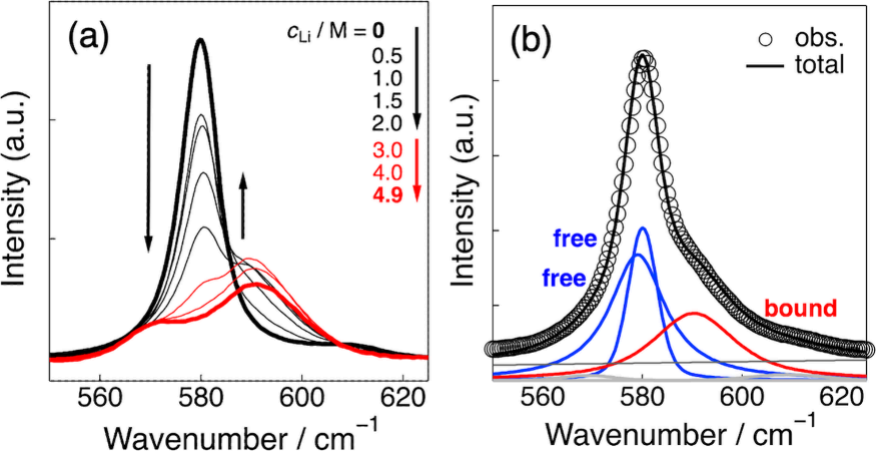
(a) Raman spectra of LiFSA/DMS electrolytes with varying Li salt concentrations (*c*_Li_). The downward and upward arrows indicate the decrease and increase in the peak intensities of the free and bound species, respectively, with increasing *c*_Li_ in the low-concentration region (0–2.0 M). (b) Typical peak-deconvolution result for the Raman spectrum of the 1.0 M LiFSA/DMS electrolyte.

Peak deconvolution analysis of the observed Raman spectra revealed that, in the concentration range of *c*_Li_ ≤ 2.0 M, the spectra can be reproduced by two components for free DMS and one component for bound DMS (Figure 1b shows a typical result for *c*_Li_ = 1.0 M). Based on eq 1, the dependence of the integrated intensity of the free-DMS peak (*I*_f_) on *c*_Li_ was analyzed to determine the average solvation number of DMS molecules around each Li^+^ ion (*n*_DMS_). The resulting *I*_f_/*c*_T_ vs *c*_Li_/*c*_T_ plot exhibited a good linear relationship as shown in Figure 2a, and the solvation number calculated from the slope and intercept was *n*_DMS_ = 2.9 ± 0.3. The ratio of the Raman scattering coefficients was determined to be *J*_b_/*J*_f_ = 1.01. The resulting *n*_DMS_ value indicates that Li^+^ ions in DMS are solvated by approximately three DMS molecules, forming a [Li­(DMS)_3_]^+^ complex. In general, Li^+^ ions in solution are known to form tetrahedrally four-coordinated complexes (*n* = 4),^37^ and similar Raman analysis in the cyclic ES solvent yielded *n*_ES_ = 4.2 in our previous study.^12^ Therefore, the flexible molecular structure of DMS, which allows conformational rearrangement, tends to decrease the solvation number compared with typical solvents, implying that the molecular flexibility of DMS plays a key role in reducing the solvation number and yielding less stable Li^+^ solvation complexes.

**Figure 2:**
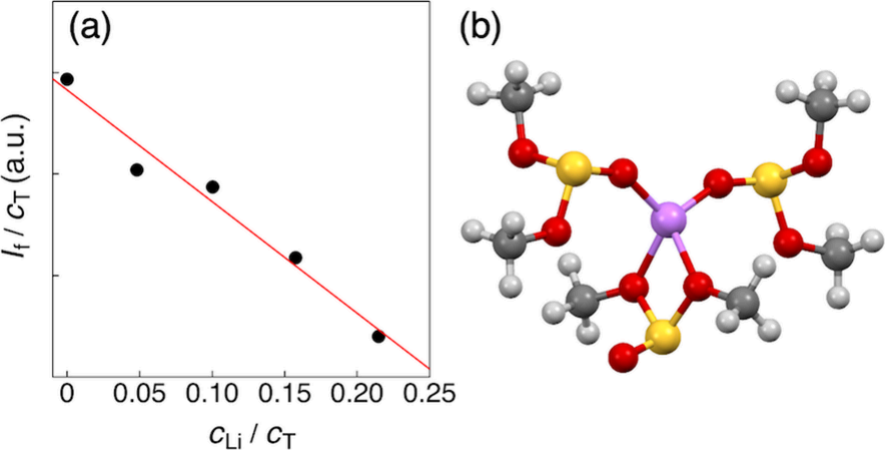
(a) *I*_f_/*c*_T_ vs *c*_Li_/*c*_T_ plot obtained from the deconvoluted Raman bands of free DMS. (b) Optimized structure of the [Li­(DMS)_3_]^+^ complex obtained from DFT calculations.

To gain further insight into the Li^+^ solvation structure, DFT calculations were performed for the [Li­(DMS)_3_]^+^ complex. The optimized lowest-energy structure is shown in Figure 2b. As seen in the optimized structure, the Li^+^ ion forms a four-coordinate complex stabilized via the oxygen atoms of three DMS molecules, involving two monodentate DMS molecules coordinated through a sulfonyl oxygen (GT conformers) and one bidentate DMS molecule coordinated through two methoxy oxygens (trans–trans (TT) conformer). According to previous DFT calculations,^27^ the TT conformer of DMS is energetically less stable by approximately 12 kJ mol^–1^ than the GG and GT conformers, suggesting that the TT form is scarcely populated in bulk solution, where GG and GT are the predominant species. The present result indicates that, within the Li^+^ solvation shell, the strong electrostatic perturbation exerted by the Li^+^ ion can stabilize the TT conformer as a thermodynamically accessible species. This finding implies that the internal energy landscape of DMS in the Li^+^ solvation environment differs markedly from that in the bulk phase, where only weak intermolecular interactions among DMS molecules are present. Notably, similar reversals in conformational stability between the Li^+^ solvation shell and the bulk phase have also been reported for other highly flexible organic solvents, such as keto ester–based solvents, amide solvents, and ionic liquids in which imide-based anions coordinate to Li^+^.^17,19,23^ The binding energy (Δ*E*_bind_) of this complex was calculated to be −444.9 kJ mol^–1^, which is approximately 10 kJ mol^–1^ less stable than that of the Li­(ES)_4_^+^ complex (*n*_ES_ = 4, Figure S2) in the cyclic ES solvent. In addition to the lowest-energy structure shown in Figure 2b, DFT calculations also identified another thermodynamically accessible Li­(DMS)_3_^+^ complex composed of two monodentate DMS molecules and one bidentate DMS molecule, all adopting the GG conformer (Figure S2). Although this GG-based complex is less stable by 20.9 kJ mol^–1^, its presence highlights that multiple coordination motifs arising from the intrinsic conformational flexibility of DMS are energetically feasible within the Li^+^ solvation environment.

In the concentrated region (3.0–4.0 M), *n*_DMS_ decreased linearly with increasing *c*_Li_, reaching as low as 1.3 at the highest concentration (*c*_Li_ = 4.9 M, Figure S3). The *n*_DMS_ value in this concentration range is nearly identical to the LiFSA:DMS molar ratio of the corresponding electrolyte samples (Table S1), indicating that all DMS molecules are coordinated to Li^+^ ions. The solution structures of highly concentrated electrolytes, particularly the coordination environment around Li^+^ ions, have been widely investigated, and it is generally established that (1) all solvent molecules and counteranions coordinate to Li^+^ ions, and (2) Li^+^ ions form ionically ordered structures (multinuclear Li^+^ complexes) interconnected through counteranions. The concentrated LiFSA/DMS system in this study exhibits similar structural characteristics, forming ionically ordered networks composed of both DMS molecules and FSA anions, which are highly analogous to those observed in the concentrated LiFSA/ES system.^12^

### Ionic Conductivity

Figure 3 shows the ionic conductivity (σ) and viscosity (η) of the LiFSA/DMS electrolytes as a function of Li salt concentration (*c*_Li_). The σ values increased with increasing *c*_Li_, reaching a maximum at approximately 1.0 M, and then decreased at higher concentrations. In contrast, the η values increased gradually up to around 2.0 M and showed a sharp increase beyond this concentration. The increase in ionic conductivity up to 1.0 M is attributed to the increase in ion concentration, whereas the decrease in σ at *c*_Li_ > 1.0 M results from the rise in viscosity, i.e., the reduction in ion mobility. These variations of σ and η with *c*_Li_ are consistent with those observed for the LiFSA/ES electrolyte system.^12^ Figure 4a,b show the temperature dependence of the σ for the dilute (1.0 M) and highly concentrated (4.9 M) LiFSA/DMS electrolytes, respectively. For comparison, the corresponding σ data for the LiFSA/ES electrolyte system are also included. In the dilute electrolytes (1.0 M), a clear difference was observed between the DMS and ES systems: throughout the measured temperature range, σ values were higher for the DMS system than for the ES system, and their temperature dependences, particularly in the high-temperature region (≥320 K), differed markedly. In contrast, in the highly concentrated electrolytes (4.9 M), both the DMS and ES systems exhibited nearly identical σ values, with little dependence on temperature, which is in sharp contrast to the behavior observed in the dilute systems. This difference is attributed to the distinct coordination structures of Li^+^ ions in the two electrolyte systems, that is, in the dilute region, Li^+^ ions are solvated by three DMS molecules in the DMS system and by four ES molecules in the ES system, whereas in the highly concentrated region, both systems form similar ionically ordered structures. Such differences in Li^+^ coordination environment are also reflected in the electrochemical behavior at the graphite negative electrode, as discussed later.

**Figure 3:**
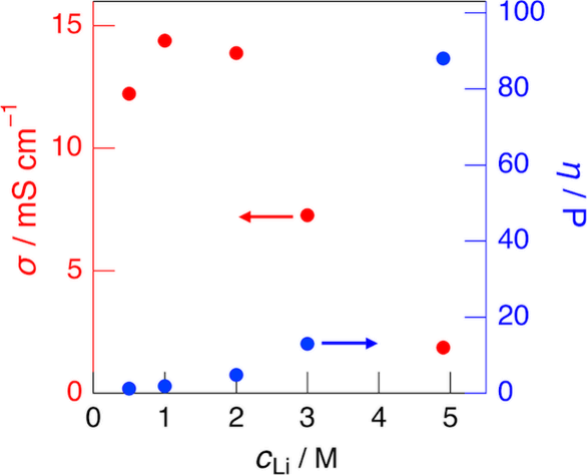
Ionic conductivity (red, left axis) and viscosity (blue, right axis) for the LiFSA/DMS electrolytes with varying Li salt concentration (*c*_Li_) at 298 K.

**Figure 4:**
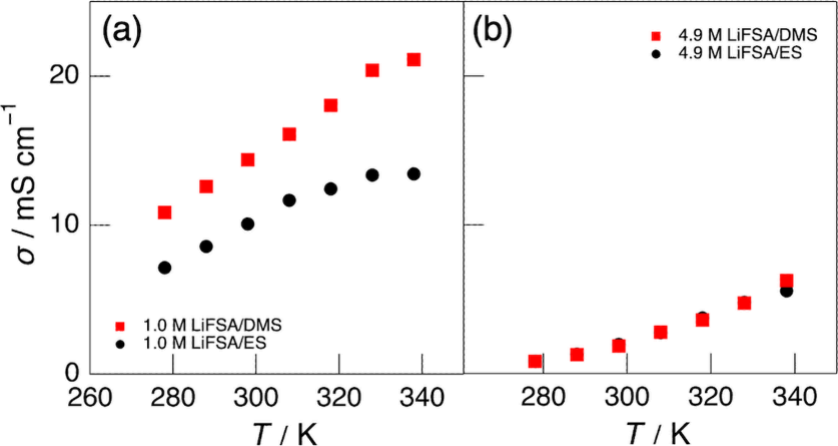
Ionic conductivity (σ) of (a) 1.0 M and (b) 4.9 M LiFSA/DMS (red squares) and LiFSA/ES (black circles) electrolytes as a function of temperature.

### Graphite Electrode Reaction

Figure 5 shows the cyclic voltammograms (CVs) of the graphite negative electrode in the dilute (1.0 M) and highly concentrated (4.9 M) LiFSA/DMS electrolytes. In both electrolytes, a clear reduction current was observed around 0.3–0 V (vs Li/Li^+^) during the cathodic scan, corresponding to Li^+^ insertion into the graphite electrode, followed by an oxidation current around 0–0.4 V during the anodic scan, associated with Li^+^ deinsertion. The observed redox peaks were split into multiple components, which can be attributed to the sequential formation of stage structures, i.e., Li–graphite intercalation compounds.^6,7,38,39^ In the dilute electrolyte, the current response gradually decreased with repeated cycles; indeed, the corresponding charge–discharge tests of the graphite/Li half-cells exhibited poor cycling performance (as discussed later). In contrast, in the highly concentrated electrolyte, the CV profiles remained stable with repeated cycling. This difference is attributed to the distinct solid–electrolyte interphase (SEI) films formed on the graphite electrode in the dilute and highly concentrated electrolytes. Regarding SEI formation, in the dilute electrolyte, a small broad reduction current was observed around 2.0 V during the first cathodic scan, which is attributed to the reductive decomposition of DMS and the consequent formation of a DMS-derived SEI film.^40,41^ In contrast, in the highly concentrated electrolyte, the reduction current around 2.0 V nearly disappeared, while a new reduction current appeared at approximately 1.0–1.5 V. This feature is commonly observed in highly concentrated electrolytes composed of FSA anions and is attributed to the reductive decomposition of FSA anions, that is, the formation of an FSA-derived SEI film.^42,43^ These results suggest that the composition of the SEI film formed on the graphite electrode strongly depends on the Li salt concentration: the DMS-derived SEI film formed in the dilute electrolyte is less stable than the FSA-derived SEI film formed in the highly concentrated electrolyte, which may partly explain the difference in cycling stability depending on the Li salt concentration.

**Figure 5:**
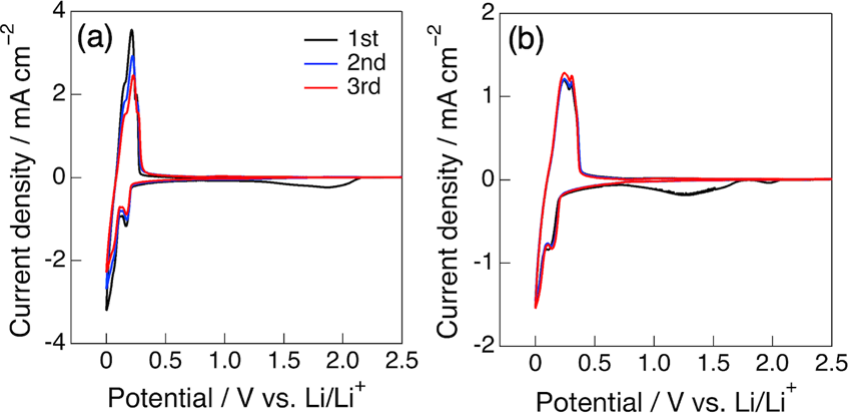
Cyclic voltammograms (CVs) of graphite electrodes in (a) 1.0 M and (b) 4.9 M LiFSA/DMS electrolytes with a scan rate of 0.1 mV s^–1^.

Figure S4 shows the potential-dependent impedance spectra (Nyquist plots) of the graphite negative electrode measured in LiFSA/DMS electrolytes at *c*_Li_ = 1.0 and 4.9 M, together with the corresponding data for the LiFSA/ES electrolyte system for comparison. In all electrolyte systems, a semicircle-like feature systematically appears and becomes more pronounced as the electrode potential approaches 0.1 V (vs Li/Li^+^). This clear potential dependence indicates that the low-frequency impedance response is directly associated with the Li^+^ insertion process at the graphite electrode, rather than originating solely from surface film resistance or measurement artifacts. Such behavior is consistent with previous studies reported for conventional carbonate-based electrolyte systems.^3,5,44^ Figure S5 shows the temperature-dependent Nyquist plots observed for the graphite electrode at a fixed potential of 0.1 V in LiFSA/DMS electrolytes with *c*_Li_ = 1.0 and 4.9 M, together with those for the LiFSA/ES electrolyte system. In all systems, the diameter of the semicircle observed in the low-frequency region decreases monotonically with increasing temperature. By fitting the impedance spectra with an equivalent circuit model, the resistance component appearing in the low-frequency region was extracted. Hereafter, this resistance is denoted as the low-frequency resistance (*R*_LF_), which reflects interfacial Li^+^ insertion processes with contributions from diffusion-related phenomena. The temperature dependence of the obtained *R*_LF_ values (Arrhenius plots) is shown in Figure 6a,b. According to the Arrhenius equation, 1/*R*_LF_ ∝ *A* exp­(−*E*_a_app_/*RT*), where *A* is the pre-exponential factor and *R* is the gas constant, the slope of the plot provides the apparent activation energy (*E*_a_app_) for the interfacial Li^+^ insertion process. In the dilute electrolytes (1.0 M, Figure 6a), the slopes of the Arrhenius plots differed significantly between the DMS and ES systems, and the *E*_a_app_ were estimated to be 22.0 ± 1.2 and 49.0 ± 3.8 kJ mol^–1^, respectively. This result indicates that the electrode reaction in the DMS system is kinetically more favorable than that in the ES system, exhibiting a remarkably lower *E*_a_app_ value even compared with that of conventional carbonate-based electrolytes (*c*_Li_ = 1.0 M, 53–59 kJ mol^–1^).^2–5^ As discussed earlier, in the dilute DMS electrolyte, the molecular flexibility of DMS leads to the formation of energetically less stable Li^+^ solvation complexes with a smaller solvation number, composed of two monodentate DMS (GT conformers) and one bidentate DMS (TT conformer). Consequently, Li^+^ desolvation during the electrode reaction proceeds more readily, which contributes to a significantly lower *E*_a_app_, as reflected in the low-frequency resistance (*R*_LF_). In contrast, in the dilute ES electrolyte, Li^+^ ions form conventional Li­(ES)_4_^+^ complexes (solvation number of four, all monodentate ES molecules), and the desolvation process strongly depends on the Li–solvent interactions. For monodentate solvents, the binding energy between Li^+^ and solvent molecules, often correlated with the Gutmann donor number, determines the energy required for bond dissociation; thus, a stronger Li–solvent interaction leads to a higher *E*_a_app_. In the highly concentrated electrolytes, both the DMS and ES systems exhibited nearly identical linear relationships (Figure 6b), and the obtained *E*_a_app_ values were 37.0 ± 1.8 and 34.6 ± 2.0 kJ mol^–1^, respectively. This similarity suggests that, in the highly concentrated region, both electrolyte systems form analogous ionically ordered Li^+^ structures (ionic aggregates), in which Li^+^ ions are interconnected via FSA anions. In such a regime, the main interaction component is the Li^+^–FSA^–^ ionic interaction, and the desolvation process governing the activation energy is predominantly influenced by this ion–ion interaction. Therefore, the destabilization effect of the Li^+^ solvation complex induced by the molecular flexibility of DMS (i.e., weak bidentate coordination) becomes much less significant in the highly concentrated electrolytes, where the electrode kinetics are instead controlled by the Li^+^–FSA^–^ interactions.

**Figure 6:**
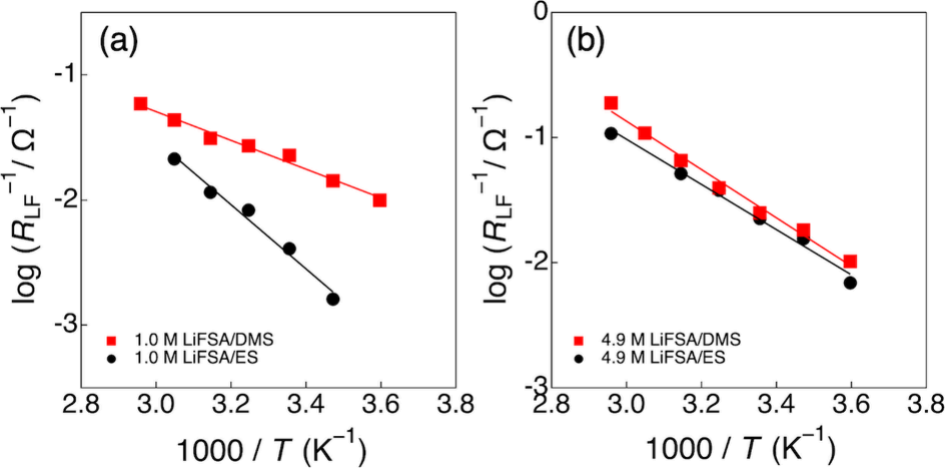
Arrhenius plots of the inverse low-frequency resistance (1/*R*_LF_) for graphite electrodes in (a) 1.0 M and (b) 4.9 M LiFSA/DMS (red squares) and LiFSA/ES (black circles) electrolytes.

Finally, to evaluate the applicability of the LiFSA/DMS electrolytes as electrolytes for Li-ion batteries, charge–discharge tests were conducted using graphite/Li half cells (Figure S6). Figure 7 shows the cycling performance of the discharge capacity and Coulombic efficiency for the dilute (1.0 M) and highly concentrated (4.9 M) LiFSA/DMS electrolytes. In the dilute electrolyte (Figure 7a), the initial discharge capacity was comparable to the theoretical capacity of graphite (372 mAh g^–1^); however, the capacity significantly degraded after several cycles, reaching 21.1 mAh g^–1^ (5.5% of the initial capacity) after 50 cycles. This poor cycling stability is most likely due to the inferior quality of the DMS-derived SEI film formed in the dilute electrolyte, which fails to suppress the reductive decomposition of the electrolyte, as discussed above. In contrast, the highly concentrated electrolyte (Figure 7b) exhibited much better cycling stability, retaining a discharge capacity of 319 mAh g^–1^ (86.7% of the initial capacity) after 50 cycles, with the Coulombic efficiency remaining close to 100%.

**Figure 7:**
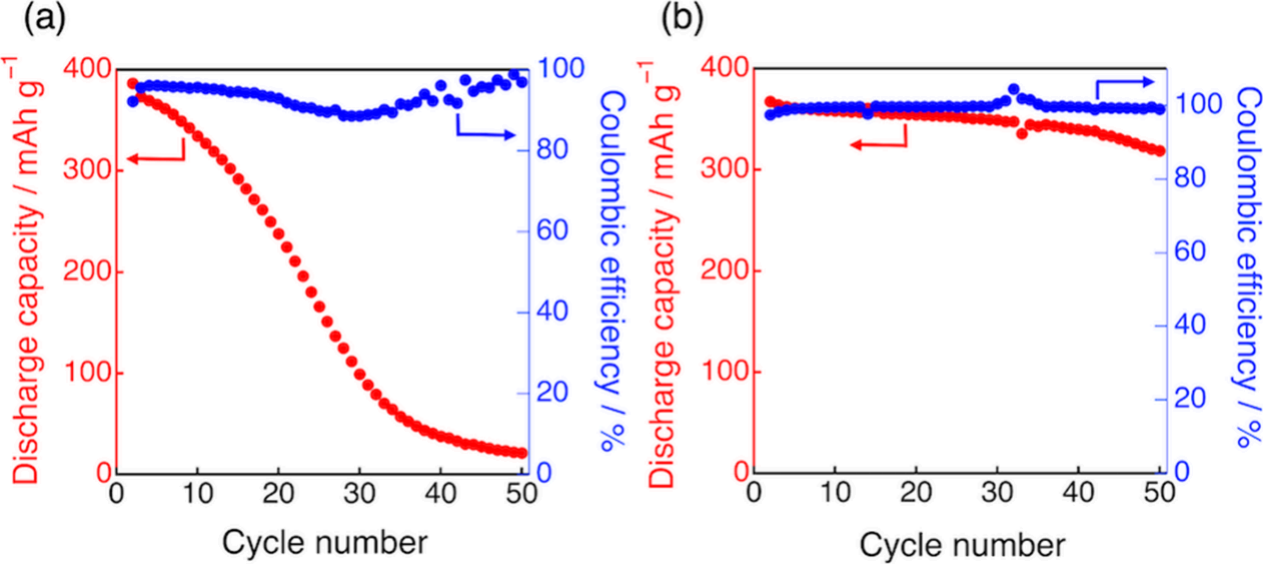
Cycling performance of graphite/Li half cells using (a) 1.0 M and (b) 4.9 M LiFSA/DMS electrolytes. Discharge capacity (red circles, left axis) and Coulombic efficiency (blue circles, right axis) are plotted as a function of cycle number. Measurements were conducted at 298 K under a constant current density of 0.16 mA cm^–2^.

Based on these results, we conclude that although DMS exhibits kinetically favorable electrode reaction characteristics as a solvent for Li-ion battery electrolytes, it suffers from a critical issue in terms of reductive stability. To address this problem, the formation of a stable SEI film is indispensable. One effective approach to achieving this is the use of a highly concentrated electrolyte, which, as demonstrated in this study, significantly improves cycling stability. To develop practical DMS-based electrolytes for Li-ion batteries, the introduction of suitable additives capable of forming a stable SEI film under dilute conditions, where the activation energy remains remarkably low, will be essential. For example, mixed electrolytes composed of DMS and ES, which share similar chemical structures and in which ES is known to facilitate the formation of a stable SEI, could be promising candidates. Elucidating the preferential Li^+^ solvation behavior as a function of the DMS/ES mixing ratio from a structural perspective may enable the realization of Li-ion battery systems that combine kinetically favorable electrode reactions with long-term cycling stability.

## Conclusions

This study elucidated how the molecular flexibility of a linear sulfite solvent, dimethyl sulfite (DMS), governs both the Li^+^ solvation structure and the electrode reaction kinetics in Li-ion battery electrolytes. Raman spectroscopy coupled with quantitative analysis revealed that, in the dilute region, Li^+^ ions form energetically less stable three-coordinate Li­(DMS)_3_^+^ complexes, due to the inherent conformational flexibility of DMS molecules, whereas the cyclic sulfite solvent ES stabilizes conventional four-coordinate Li­(ES)_4_^+^ complexes. DFT calculations further demonstrated that the strong electrostatic field of Li^+^ can stabilize the otherwise unfavorable TT conformer of DMS within the solvation shell. In the highly concentrated region, both DMS and ES formed analogous ionically ordered structures interconnected through FSA anions. These structural features directly impacted the electrode reaction kinetics. The dilute DMS electrolyte exhibited an exceptionally low activation energy for the Li^+^ insertion reaction at the graphite electrode, a consequence of the easier desolvation of the weakly coordinated Li^+^ species. However, this kinetic advantage was accompanied by poor reductive stability, resulting in unstable DMS-derived SEI films and severe capacity degradation. In contrast, the highly concentrated DMS electrolyte yielded stable FSA-derived SEI films and markedly improved cycling stability, although the activation energy increased due to the dominance of Li^+^–anion interactions within the ionically ordered structures. Overall, this study demonstrates that the molecular flexibility of the solvent is a key factor controlling the Li^+^ coordination environment and interfacial reaction kinetics in Li-ion battery electrolytes.

## Supplementary Material



## Notes

*c*_Li_, molar ratio, density for LiFSA/DMS solutions (Table S1); theoretical Raman bands for isolated DMS (Figure S1); optimized geometry and Δ*E*_bind_ values for Li-solvent complexes (Figure S2); peak deconvolution and *n*_DMS_ vs *c*_Li_ plot in concentrated region (Figure S3); potential-dependent impedance spectra (Figure S4); temperature-dependent impedance spectra (Figure S5); charge–discharge profiles (Figure S6) (PDF)
