# Supplementary material for: Molecular Flexibility-Controlled Ion Solvation and Electrode Reaction Kinetics in Sulfite-Based Lithium-Ion Battery Electrolytes
Source: J Phys Chem B. 2026 Feb 19;130(9):2619–26. doi: 10.1021/acs.jpcb.5c08092 (PMC12969258; doi:10.1021/acs.jpcb.5c08092)
Supplement: Supplementary file 1 [file jp5c08092_si_001.pdf]

Supporting Information

Molecular Flexibility-Controlled Ion Solvation and Electrode Reaction Kinetics in Sulfite-Based Lithium-Ion Battery Electrolytes

*Misa Yamashita,[a] Saki Sawayama,[a] and Kenta Fujii [a]**

[a] *Graduate School of Sciences and Technology for Innovation, Yamaguchi University, 2-16-1 Tokiwadai, Ube, Yamaguchi 755-8611, Japan.*

Corresponding Author

*E-mail: k-fujii@yamaguchi-u.ac.jp (K.F.)

Table S1. Li salt concentration (c_{Li}), LiFSA-to-DMS molar ratio, and density (d) of LiFSA/DMS electrolyte solutions.

c_{Li} / M	LiFSA : DMS	d / g cm^{-3}
0	–	1.2063
0.5	1 : 21	1.2534
1.0	1 : 10	1.2972
1.5	1 : 6.4	1.3372
2.0	1 : 4.5	1.3765
3.0	1 : 2.7	1.4493
4.0	1 : 1.8	1.5192
4.9	1 : 1.3	1.5802

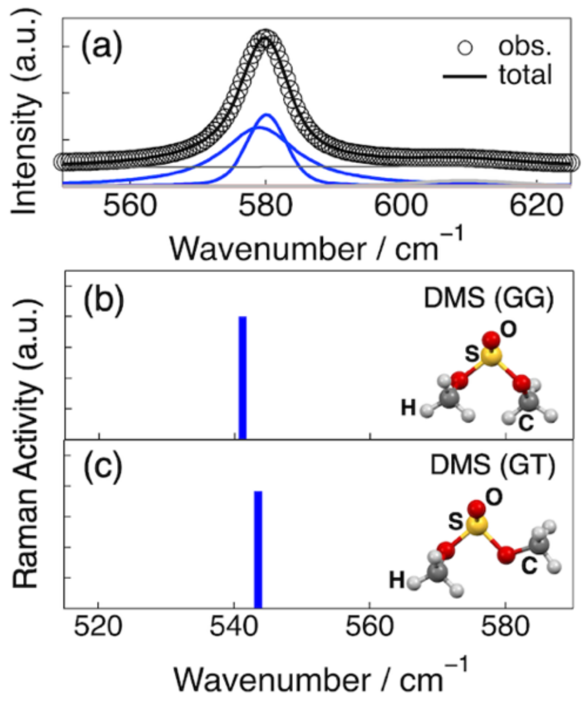

Figure S1. (a) Observed Raman spectrum of neat DMS and the theoretical Raman bands calculated for isolated DMS in the (b) GG and (c) GT conformers. The optimized geometries of each conformer obtained from DFT calculations are also shown.

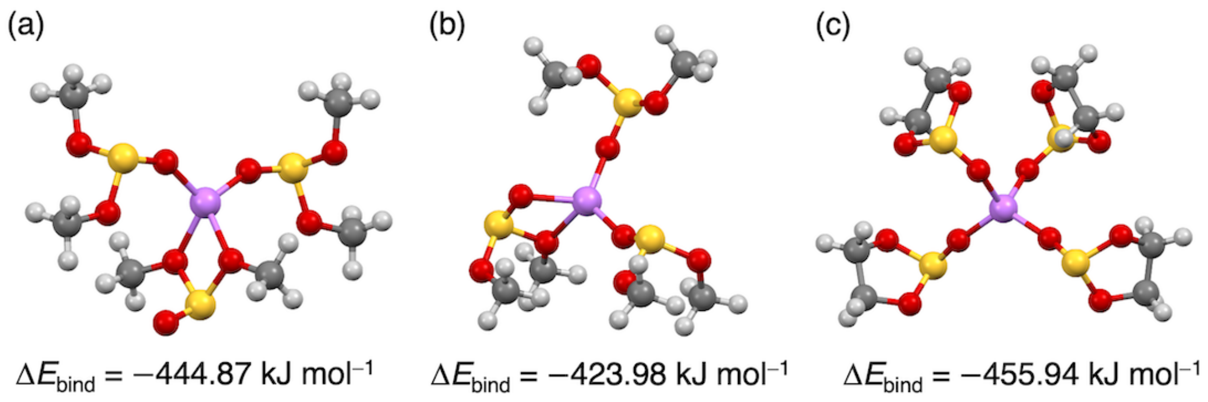

Figure S2. Optimized geometries and binding energies (ΔE_{bind}) of Li^+ complexes obtained from DFT calculations: (a) $Li(DMS)_3^+$ complex containing two monodentate GT conformers and one bidentate TT conformer, (b) $Li(DMS)_3^+$ complex containing two monodentate GG conformers and one bidentate GG conformer, and (c) $Li(ES)_4^+$ complex.

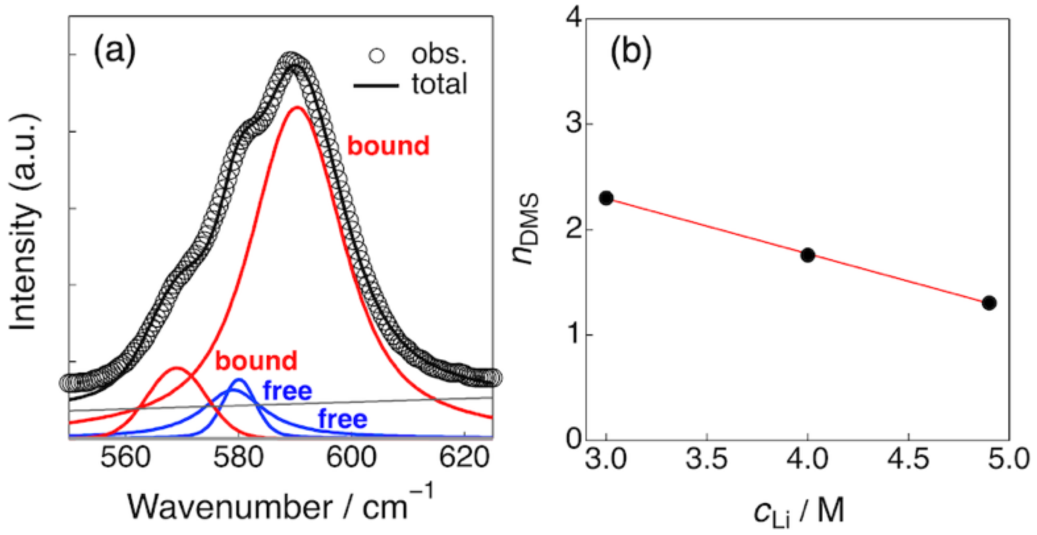

Figure S3. (a) Typical peak-deconvolution result for the Raman spectrum of the 3.0 M LiFSA/DMS solution. (b) Dependence of the solvation number (n_{DMS}) on c_{Li} for LiFSA/DMS electrolyte solutions.

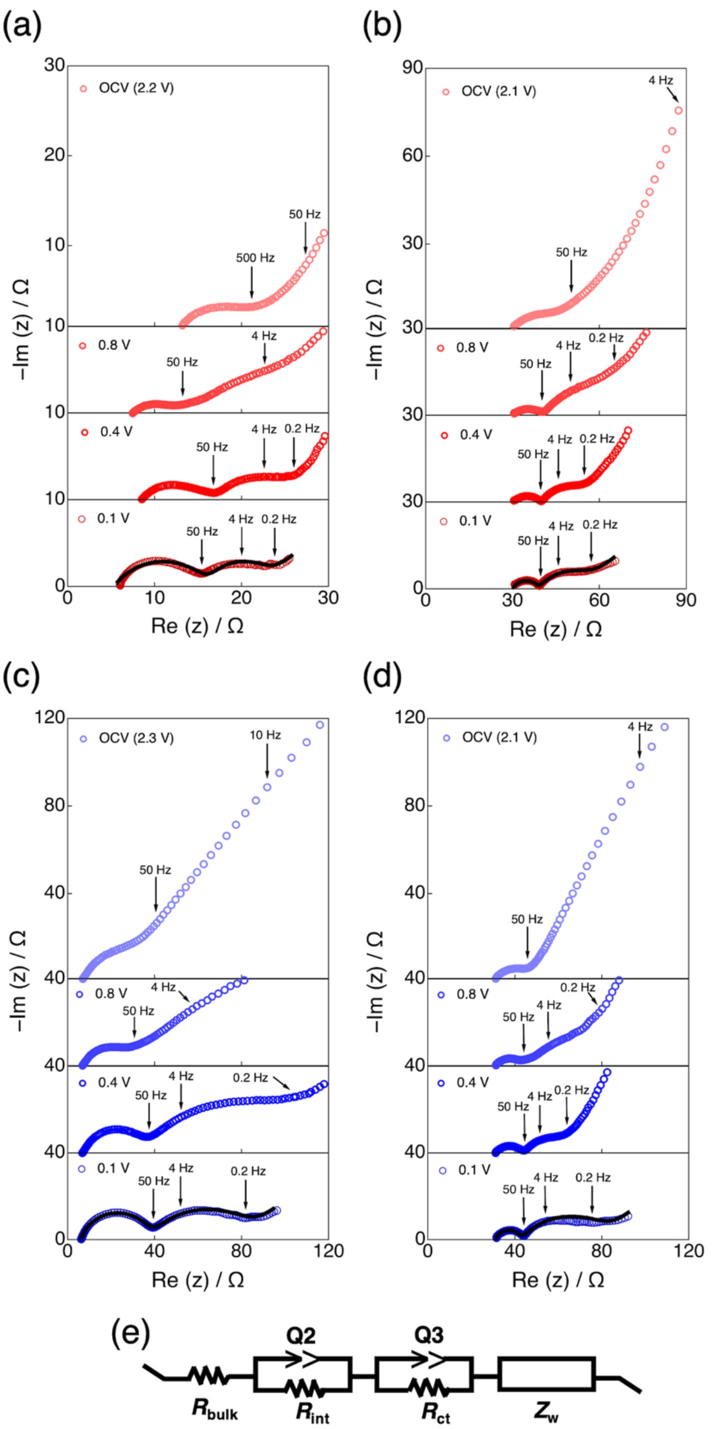

Figure S4. Potential-dependent Nyquist plots for the graphite electrode in (a) 1.0 M LiFSA/DMS, (b) 4.9 M LiFSA/DMS, (c) 1.0 M LiFSA/ES, and (d) 4.9 M LiFSA/ES electrolytes at 298 K. The impedance spectra were recorded at various electrode potentials, including the open-circuit voltage (OCV) and the Li^+ insertion region down to 0.1 V (vs Li/Li^+).

For the spectra measured at 0.1 V, where Li^+ insertion dominantly occurs, quantitative fitting analysis was performed over a frequency range from 4 mHz to 1.0 MHz using an equivalent circuit model (shown in panel (e)) that includes a diffusion-related Warburg impedance element in the low-frequency region. The fitting results are shown as solid black lines.

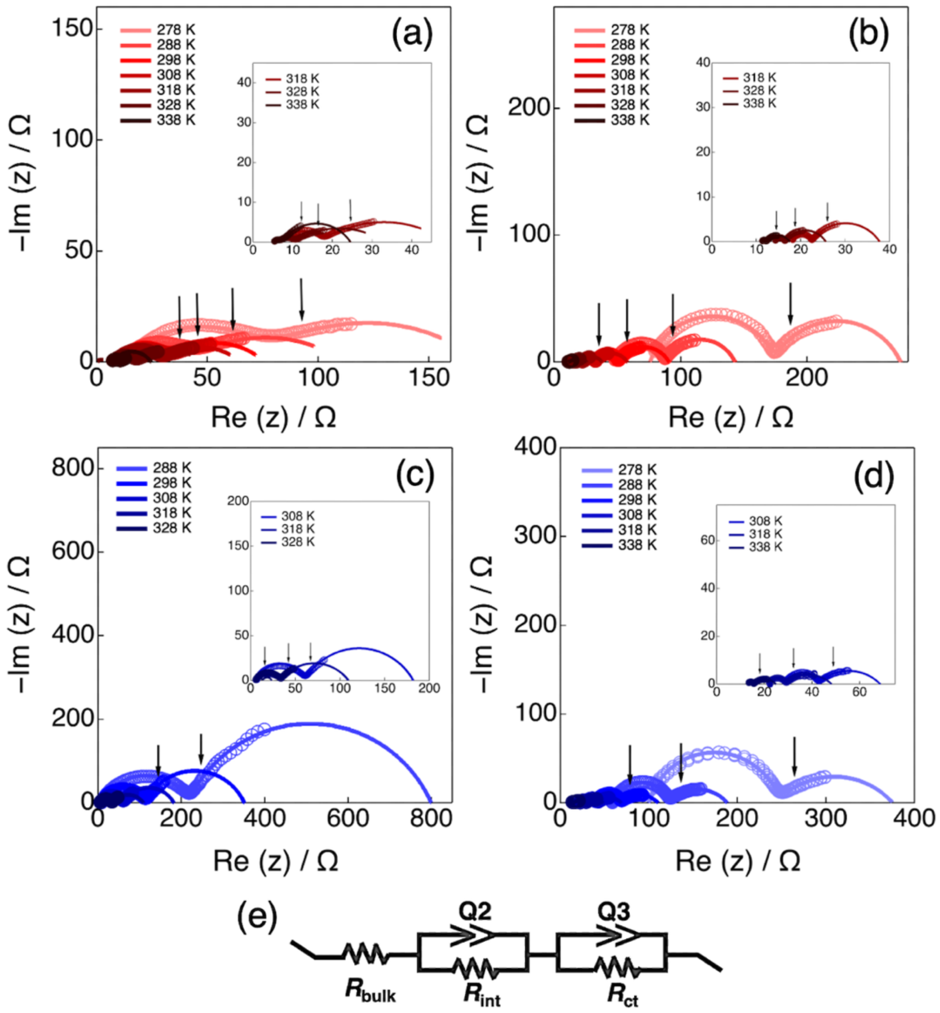

Figure S5. Nyquist plots of graphite electrodes measured at various temperatures (278–338 K) in (a) 1.0 M LiFSA/DMS, (b) 4.9 M LiFSA/DMS, (c) 1.0 M LiFSA/ES, and (d) 4.9 M LiFSA/ES electrolytes. The electrode potential was fixed at 0.1 V (vs Li/Li^+). Impedance spectra were recorded over a frequency range from 20 mHz to 1.0 MHz. Solid lines represent the results of fitting using the equivalent circuit model shown in panel (e). Arrows indicate the characteristic frequency region (approximately 4 Hz) associated with the Li^+ insertion process at graphite electrodes.[1]

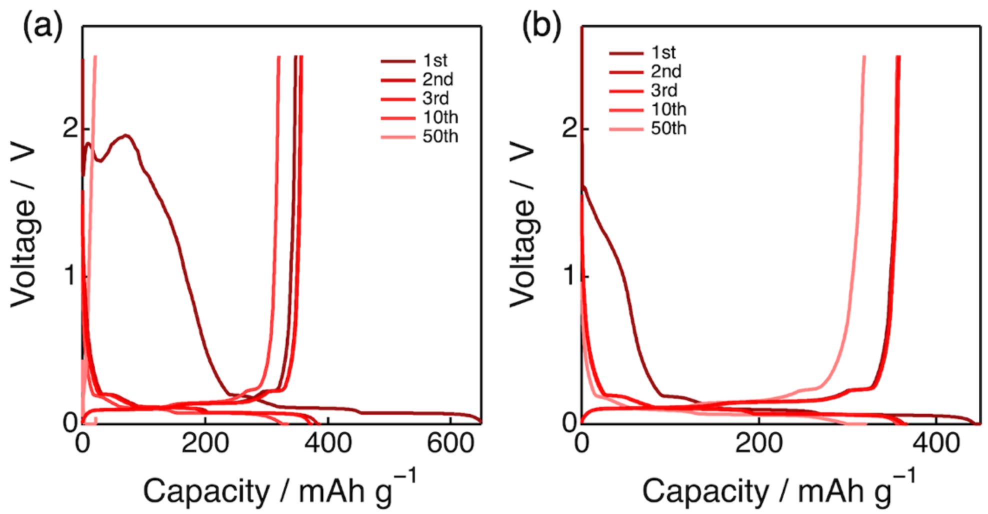

Figure S6. Typical charge–discharge profiles of graphite electrodes measured in (a) 1.0 M and (b) 4.9 M LiFSA/DMS electrolytes at the 1st, 2nd, 3rd, 10th, and 50th cycles. Measurements were performed under the same conditions as those used for the cycling performance shown in Figure 7. For clarity, the cycling performance shown in Figure 7 in the main text is plotted from the second cycle onward.

Reference

(1) Kondo, Y.; Abe, T.; Yamada, Y. Kinetics of Interfacial Ion Transfer in Lithium-Ion Batteries: Mechanism Understanding and Improvement Strategies. *ACS Appl. Mater. Interfaces* **2022,** *14*, 22706-22718.
